# Indoor air pollution in kindergartens is a stronger predictor of preschool wheezing than household pets and passive smoking

**DOI:** 10.3389/fpubh.2026.1835292

**Published:** 2026-05-29

**Authors:** Vaida Taminskiene, Nina Prokopciuk, Mindaugas Butikis, Lukas Vaidelys, Karolina Sceliokiene, Olga Bielousova, Edita Poluzioroviene, Rimantas Stukas, Arunas Valiulis

**Affiliations:** 1 Department of Public Health, Faculty of Medicine, Institute of Health Sciences, Vilnius University, Vilnius, Lithuania; 2 Human Ecology Interdisciplinary Research Group, Faculty of Medicine, Vilnius University, Vilnius, Lithuania; 3 Clinic of Children's Diseases, Faculty of Medicine, Institute of Clinical Medicine, Vilnius University, Vilnius, Lithuania

**Keywords:** indoor air pollution, kindergartens, particulate matter, preschool children, wheezing

## Abstract

**Background:**

The quality of air in kindergartens, where children spend a significant part of their time, is an important determinant of health.

**Objective:**

This study aimed to explore the relationship between indoor air pollution in kindergartens and wheezing syndrome in preschool children.

**Methods:**

Air pollution, specifically the concentration of particulate matter (PM), was measured in 24 kindergartens. A total of 1,794 parents of preschool children were surveyed about respiratory symptoms and environmental factors affecting their children.

**Results:**

The mean age of the children was 4.07 (± 1.44) years, and 50.9% were males. Wheezing history was reported in 5.8% of children, while separately evaluated physician-diagnosed bronchiolitis in 11.1% and asthma in 2.6%. One third of children were exposed to smoking and 44.2% had pets at home. Allergic rhinitis and eczema, having siblings, higher concentrations of PM_2.5_ and PM_1_ in kindergarten environments, and proximity to railways were independently associated with an increased risk wheezing of wheezing in preschool children.

**Conclusion:**

The association between wheezing and indoor PM_2.5_ and PM_1_ concentration in kindergartens outweighed other common household exposures, such as pet ownership and secondhand tobacco smoke. Therefore, improving indoor air quality through effective control of air pollutants, installation of vegetative barriers near kindergartens that promote particulate matter dry deposition from outdoor air pollution sources, and adherence to indoor air quality guidelines are essential for preventing recurrent wheeze in early childhood.

## 1 Introduction

Indoor air pollution in kindergarten environments represents a significant and often underrecognized risk to preschool children's health (1). Young children are more vulnerable to the harmful effects of air pollution compared to adults due to physiological immaturity, higher breathing rates, and behavioral factors such as more time engaged in physical activity, with exposure increasing the risk of respiratory conditions such as lower respiratory infections, asthma, and impaired lung function (2–9). Indoor air quality is of particular importance, as it is influenced not only by outdoor air pollution that infiltrates buildings, but also by a variety of pollutants generated from indoor sources, including cooking, heating, building materials, and cleaning products (10, 11). Maintaining good air quality in the kindergartens is especially critical for children, who spend the majority of their time indoors, making them more susceptible to prolonged and cumulative exposure to these contaminants, which can have significant implications for their respiratory health, immune system and overall development (12–14). Furthermore, emerging evidence suggests that poor indoor air quality may also negatively affect cognitive function, attention, and learning outcomes in early childhood (15), underscoring the importance of implementing effective air quality management strategies in early education environments. Respiratory symptoms, including wheezing, prolonged cough, dry nighttime cough without an accompanying cold, and shortness of breath, are relatively common among children and may occur even in those without a formal diagnosis of asthma (16), indicating that many children without a formal diagnosis may still experience significant respiratory symptoms. The presence of these symptoms in undiagnosed children underscores the critical importance of environmental factors in respiratory health (16, 17). Exposure to environmental pollutants such as particulate matter, volatile organic compounds, nitrogen dioxide, tobacco smoke, indoor mold, and allergens can irritate and inflame the airways, triggering wheezing and other respiratory symptoms (18, 19). Poor air quality, both indoors and outdoors, can contribute to airway hyper responsiveness and reduced lung function, increasing susceptibility to respiratory distress (20–22). Therefore, the high prevalence of respiratory symptoms among children highlights the need to consider environmental quality as a key determinant of respiratory health.

Although most preschool-aged children attend kindergartens and spend a substantial portion of their day in these environments (23), there is a limited amount of research examining indoor air quality in such settings and its implications for children's respiratory health. While the associations between air pollution and respiratory symptoms are well established and extensively studied in general population (2) and among older children (16, 24, 25), moreover, evidences exists regarding the relationship between outdoor air pollution and respiratory symptoms in preschool-aged children (26), there is a notable lack of research specifically addressing air quality within kindergarten environments and its relationship with wheezing syndrome prevalence among preschool children. Furthermore, existing studies have primarily focused on measuring pollutant levels in kindergartens, often without investigating their direct links to respiratory outcomes (3, 27, 28). This gap in research underscores the need for more targeted and comprehensive investigations into the impact of particulate matter exposure in early childhood settings. Accordingly, the present study aims to evaluate the relationship between particulate matter exposure in kindergartens and the prevalence of wheezing syndrome among preschool-aged children.

## 2 Material and methods

A cross-sectional study was conducted to investigate the association between the prevalence of wheezing symptoms in preschool children and potential determinants, including exposure to particulate matter air pollution. The study included 1,794 preschool children from 24 kindergartens across Vilnius, with data collection carried out during the 2023–2024 cold season.

Anonymous questionnaires were completed by parents of preschool children with both, the kindergarten administration and parents' permission. The study protocol was approved by the Vilnius Regional Biomedical Research Ethics Committee (protocol code 2024/3-1575-1035).

### 2.1 Sample selection

According to data from the Vilnius City Municipality, there are 126 public kindergartens in Vilnius, attended by nearly 27,000 children aged 1 to 7 years. A total of 38 kindergartens agreed to take part in indoor air quality measurements. From these, 24 kindergartens were randomly selected, where air pollution measurements were conducted and questionnaires were distributed. In each selected kindergarten, between 80 and 120 questionnaires were distributed, depending on the size of the institution and the number of enrolled children. The number of returned questionnaires per kindergarten ranged from 35 to 105, with an average of 74.75 (±15.37) questionnaire per institution. The response rate was from 43.75 to 87.50%. In total, 1,794 fully completed questionnaires were collected. This sample represents children attending public preschool education institutions in Vilnius with an estimated 3% confidence of limit and 99% confidence level.

The sampling process was conducted with the support of the Vilnius City Public Health Bureau, which assisted in data collection and in obtaining the necessary permissions to carry out the study.

### 2.2 Particulate matter measurement

Air pollutants were measured in 24 kindergartens in Vilnius during the cold season of 2023–2024. The cold season was selected for air pollution assessment because pollutant levels are generally higher (29). Moreover, during this period, children spend more time indoors and attendance in educational institutions tends to increase, resulting in greater potential exposure.

To determine seasonal aerosol particle number concentrations in kindergartens an optical particle sizer (TSI model 3330, PNC and PMC 0.3–10.0 μm) was used. Original indoor short-term (10 min) serial measurements were carried out. Measurements of fraction particulate matter [PM_1_ (0.3-1μm), PM_2.5_ (0.3-2.5μm), PM_10_ (0.3-10μm)] were performed. Indoor measurements at the kindergartens were conducted from 9 am to 13 pm in children's sleeping rooms and playrooms, with 8–12 measurements per kindergarten, depending on its size, each lasting 10 min. The devices were placed on the children's table, away from the children, while they were playing. All measurements in each kindergarten were performed within a single day.

Aerosol particle number concentrations, rather than mass concentrations, were selected, as particle mass is predominantly determined by a small number of larger particles that are more readily cleared from the respiratory tract. In contrast, finer particles, for which no regulatory limit values have been established, remain suspended in the air for longer periods and may have a greater impact on respiratory health (7).

### 2.3 Questionnaire

The questionnaires were completed by parents and collected information on children's respiratory symptoms, including a history of wheezing, shortness of breath, and cough, as well as the presence of diagnosed asthma, atopic dermatitis, allergic rhinitis, and history of respiratory infections Parents also provided personal information about their children, such as age, gender, height, weight, and the presence of siblings. In addition, the questionnaires collected personal information and details on household environmental conditions to assess potential environmental exposures affecting children's health. Parents were asked about the type of housing, the presence of pets in the home, the type of heating (central, electricity, gas, geothermal, solid fuel, or other), and the type of cooking stoves used (gas or electric). Information was also obtained on whether the household had an air conditioning system. In addition, residential exposure to environmental pollution was evaluated. Parents reported whether their home was located near a high-traffic road or in close proximity to a railway. Children's exposure to second-hand smoke was also assessed. Parents provided information regarding smoking, including the type of cigarettes, within the household or other environments frequently attended by the child. The analysis was conducted using selected questions from a broader instrument designed to assess allergic and respiratory diseases. A Lithuanian version of the questionnaire was used in the survey.

### 2.4 Outcome measure

The European Respiratory Society's definition of wheezing was used in this study (9, 19). Children were classified as having wheezing syndrome if their parents reported at least one episode of wheezing during their lifetime, specified as a question: “Has your child ever experienced shortness of breath or wheezing in the chest?” Parents separately indicated the cases of physician-diagnosed bronchiolitis, pneumonia and asthma. Asthma diagnosis for children was reported by parents by responding to the questionnaire item: “Has your child ever been diagnosed with asthma by a physician?”

To gain a more comprehensive understanding of the impact of the kindergarten environment on children's health and wellbeing, supplementary variables were derived. For each of the 24 kindergartens participating in the study, the proportion of children who had experienced wheezing, had been diagnosed with asthma, or had a prolonged cough lasting 2 months or more was calculated. The prevalence of wheezing ever in each kindergarten was used as the primary outcome variable. Kindergartens in the highest quartile (4th quartile) of wheezing syndrome prevalence were considered to have the highest risk of wheezing and were compared with the remaining kindergartens.

**Figure 1:**
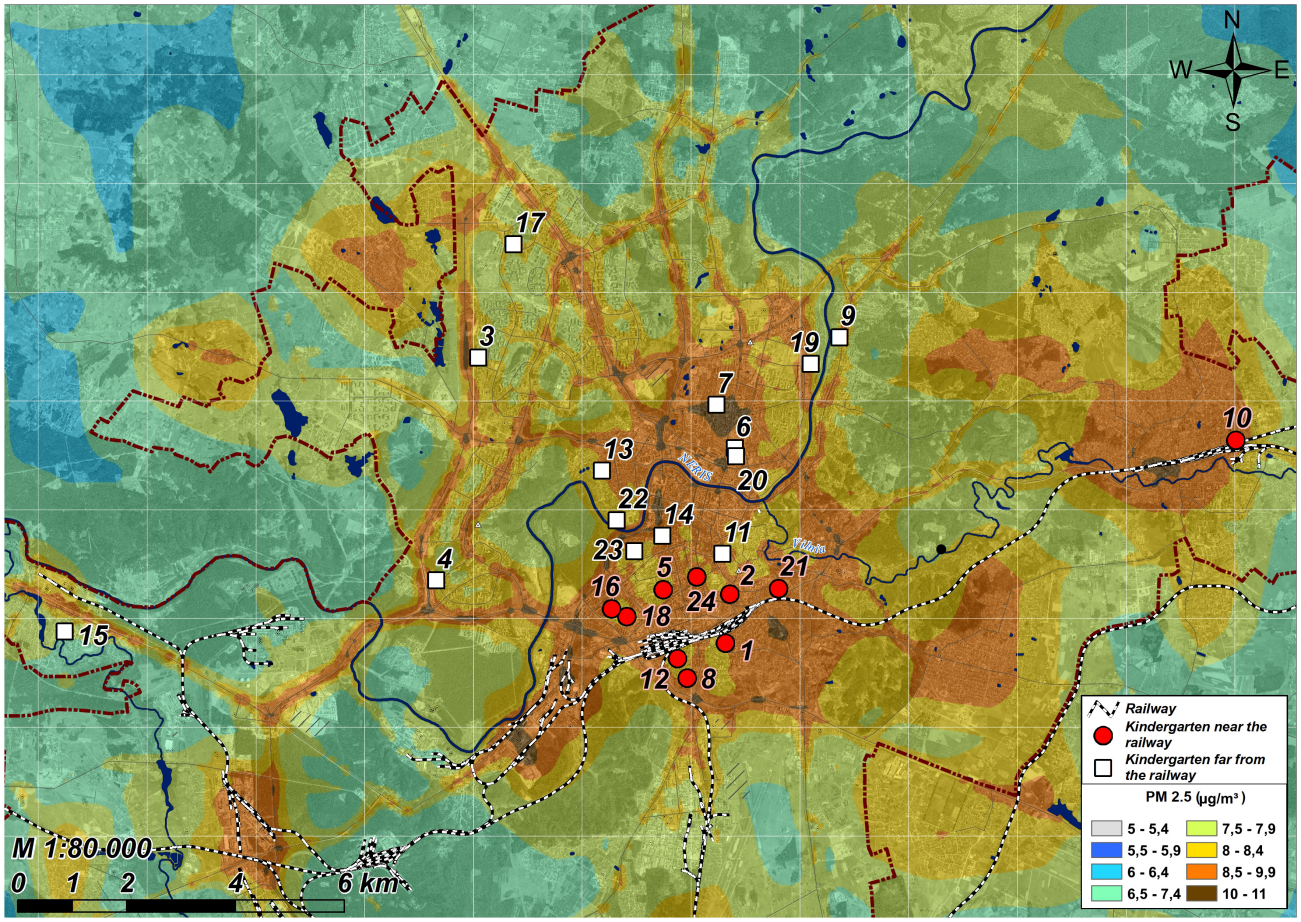
Location of kindergartens participating in the study according to proximity to the railway. Adapted from the public domain “Air Pollution Dispersion Maps” of the Environmental Protection Agency of Lithuania (https://aaa.lrv.lt/lt/veiklos-sritys/oras/kiti-oro-uzterstumo-sklaidos-zemelapiai/), published with permission.

Furthermore, kindergartens located within 1 km of a railway were classified as being in proximity to a railway (Figure 1) and compared with those situated further away. In total, 10 kindergartens attended by 44.1 % (792) of study respondents were located within 1 km of an active railway line. Although Kindergarten No. 15 is situated near a railway branch, this line is characterized by infrequent and irregular use compared with the main railway line, as it primarily serves local industrial activities rather than regular passenger traffic. Therefore, its inclusion in the group classified as being in proximity to a railway was not considered appropriate. This categorization enabled the assessment of potential associations between railway proximity and the prevalence of respiratory symptoms among children.

Although the study focuses on the kindergarten environment, children may be exposed to air pollutants not only in daycare settings but also in other environments, such as their homes or during time spent outdoors. Taking this into account, the questionnaire included items assessing potential previously mentioned environmental exposures at home. All of these variables were subsequently incorporated into the analysis.

### 2.5 Statistical analysis

Descriptive statistics, univariate and multivariate analyses were performed. Univariate analyses were used to compare differences between children with and without wheezing syndrome. The independent *t*-test was used to compare differences between two groups for normally distributed variables. The Mann–Whitney U test was used to compare differences between two independent groups for non-normally distributed variables. The chi-square test was applied to assess associations between categorical variables. When expected cell counts were small—the Fisher's exact test was employed as a more accurate alternative for evaluating associations between categorical variables. Binary logistic regression analysis was conducted to identify factors independently associated with a higher prevalence of wheezing symptoms among preschool children. Statistical analyses were performed using IBM SPSS Statistics for Windows, version 31 (IBM Corp., Armonk, NY, USA), OpenEpi and Microsoft Excel. Differences were considered statistically significant at p < 0.05.

## 3 Results

### 3.1 Characteristics of study participants

The mean age of the children was 4.07 (± 1.44) years, and 50.9% were boys. The mean duration of kindergarten attendance was 24.23 (± 15.09) months.

Approximately one-third of children were exposed to secondhand smoke, with a similar proportion exposed to emissions from combustible (conventional) cigarettes and electronic cigarettes. Overall, 20.7% of children lived within 200 meters of a high-traffic road, while the remaining children lived further away. In addition, more than 40% of respondents reported having a cat or dog in their household. Further characteristics of the respondents are presented in Table 1.

**Table 1:** Characteristics of study participants depending on the presence of wheezing syndrome.

Characteristics		Total (*N* = 1,794)	Without reported wheeze episode (*N* = 1,679)	With at least one reported wheeze episode (*N* = 98)
% Males (*n*)		50.9 (890)	50.2 (791)	67.3 (66)^*^
Mean age (± SD)		4.07 (±1.44)	4.07 (±1.44)	3.92 (±1.46)
Mean BMI (± SD)		15.52 (±2.02)	15.77 (±1.72)	15.50 (±2.01)
Mean BMI–z score (± SD)		−0.0005 (±1.00)	−0.0092 (±0.99)	0.1252 (±0.85)
% Diagnosed with asthma (*n*)		2.8 (47)	0.5 (7)	40.8 (4)^**^
% Have siblings (*n*)		61.5 (1,076)	61.1 (964)	64.3 (63)
% Exposed to secondhand smoking (*n*)		33 (572)	32.8 (514)	35.7 (35)
% Diagnosed with eczema (*n*)		16.5 (272)	16.0 (246)	26.6 (25)^*^
% Diagnosed with allergic rhinitis (*n*)		5.1 (87)	4.2 (64)	18.1 (17)^**^
% Have a cat or a dog at home (*n*)		44.2 (766)	44.4 (696)	44.9 (44)
% Live near the railway (*n*)		7.5 (130)	7.5 (117)	8.2 (8)
% Live near a major road (*n*)		20.7 (352)	20.8 (321)	14.6 (14)
% Have an air conditioner at home (*n*)		29.0 (506)	28.9 (455)	35.7 (35)
Housing type	% Private home (*n*)	31.8 (554)	32.3 (508)	32.7 (32)
	% Apartment (*n*)	68.2 (1,189)	67.7 (1,065)	67.3 (66)
Type of stove	% Electric (*n*)	70.5 (1,226)	70.1 (1,108)	73.5 (72)
	% Gas (*n*)	29.5 (513)	29.2 (462)	26.5 (26)

^*^p < 0.05; ^**^p < 0.001.

### 3.2 Prevalence of wheezing symptoms among preschool children

Ninety eight parents (5.8%) reported that their children had experienced shortness of breath and wheezing at any point in their lives. On the other hand, the prevalence of at least one cough episode lasting two months or longer in the population was 7.8%. Wheezing during the preceding 12 months was reported in 48 children (2.7%). Overall, 11.1% of children had a history of bronchiolitis, and 9.7% had pneumonia in the past year. The prevalence of physician-diagnosed bronchial asthma among children was 2.6%. Among children with a physician-confirmed diagnosis of asthma, 12.8% were classified as having moderate or severe disease. Allergic asthma was reported in 44.7% of cases.

Boys experience shortness of breath and wheezing more often during the preschool age. Wheezing was more commonly reported by parents in children with physician-diagnosed asthma, eczema, and allergic rhinitis. No other parents-reported home environment or lifestyle factors were associated with a higher prevalence of wheezing in preschool children.

Particulate matter concentrations were measured at 24 kindergartens. For each kindergarten, the prevalence of wheezing ever, physician-diagnosed asthma, and prolonged cough was calculated, with the results presented in Table 2.

**Table 2:** Particulate matter concentrations and prevalence of respiratory symptoms in children attending kindergartens.

Kinder-garten no	PM_1_, particles/cm^3^	PM_2.5_, particles/cm^3^	PM_10_, particles/cm^3^	% (*n*) children who experienced at least one episode of wheeze	% (*n*) children with cough, lasting >2 months	% (*n*) children with physician-diagnosed asthma
1	88 (± 16.6)	90 (± 16.5)	90 (± 20.5)	1.5 (1)	6.8 (4)	0 (0)
2	21 (± 1.5)	23 (± 1.5)	24 (± 1.6)	9.7 (6)	5.0 (3)	3.3 (2)
3	19 (± 7.5)	21 (± 7.8)	21 (± 7.9)	3.9 (3)	6.9 (5)	2.6 (2)
4	52 (± 6.9)	54 (± 7.2)	54 (± 6.9)	5.1 (4)	6.8 (5)	3.8 (3)
5	70 (± 34.9)	72 (± 35.7)	72 (± 35.7)	8.7 (4)	14.0 (6)	0 (0)
6	34 (± 7.6)	35 (± 7.0)	36 (± 9.4)	9.2 (1)	5.4 (4)	5.6 (4)
7	70 (± 10.1)	71 (± 10.1)	71 (± 10.2)	3.0 (2)	14.1 (9)	1.5 (1)
8	98 (± 9.5)	98 (± 9.4)	99 (± 9.4)	7.2 (6)	1.2 (1)	1.2 (1)
9	43 (± 4.1)	44 (± 3.8)	44 (± 7.8)	4.9 (3)	6.2 (4)	3.1 (2)
10	46 (± 22.3)	47 (± 22.2)	47 (± 22.9)	3.9 (3)	13.2 (10)	1.3 (1)
11	13 (± 8.6)	13 (± 9.0)	13 (± 8.6)	5.7 (4)	14.1 (10)	5.8 (4)
12	34 (± 20.0)	35 (± 20.2)	35 (± 20.2)	9.5 (7)	13.5 (10)	9.5 (7)
13	121 (± 22.5)	122 (± 22.4)	122 (± 22.3)	6.7 (4)	9.8 (6)	1.6 (1)
14	14 (± 0.8)	15 (± 0.9)	15 (± 1.0)	0 (0)	14.8 (8)	0 (0)
15	64 (± 8.8)	66 (± 8.6)	66 (± 8.6)	5.6 (5)	3.3 (3)	0 (0)
16	24 (± 3.9)	25 (± 4.0)	26 (± 4.2)	6.1 (6)	8.9 (9)	2.9 (3)
17	52 (± 47.6)	53 (± 47.8)	54 (± 47.8)	4.2 (4)	4.3 (4)	2.1 (2)
18	29 (± 4.5)	31 (± 4.7)	31 (± 5.2)	6.8 (5)	12.2 (9)	4.0 (3)
19	63 (± 12.7)	64 (± 12.6)	64 (± 12.4)	7.1 (6)	13.3 (11)	2.4 (2)
20	53 (± 22.5)	54 (± 22.8)	55 (± 22.7)	6.2 (4)	3.2 (2)	3.0 (2)
21	57 (± 16.5)	58 (± 16.2)	59 (± 15.3)	8.7 (6)	10.4 (7)	5.7 (4)
22	122 (± 14.2)	124 (± 13.5)	126 (± 13.2)	3 (1)	6.7 (2)	0 (0)
23	16 (± 4.1)	18 (± 4.5)	19 (± 4.8)	4.4 (2)	6.5 (3)	2.2 (1)
24	186 (± 323.0)	188 (± 324.0)	188 (± 324.0)	6.6 (5)	7.2 (5)	2.7 (2)
Total	57 (± 39.9)	58 (± 39.9)	59 (± 39.9)	5.8 (98)	8.5 (140)	2.8 (30)

### 3.3 Factors associated with wheezing symptoms

In order to find out the impact of the kindergarten environment on wheezing in children, the prevalence of wheezing syndrome in each of the kindergartens was estimated and varied from 0 to 9.7%. Characteristics of children attending kindergartens with the highest prevalence of parent-reported wheezing syndrome was compared with all other kindergartens (Table 3). Wheezing history was more prevalent among younger children, those with a physician-diagnosed asthma, children living in a private house rather than an apartment, and children with siblings compared to those without. Moreover, the median concentrations of all measured particulate matter fractions were significantly higher in kindergartens with a higher wheezing prevalence than in those with a lower prevalence.

**Table 3:** Characteristics of the study kindergartens in relation to the different prevalence of wheezing syndrome among the attendees of these kindergartens.

Characteristics		Total (*N* = 1,794)	Kindergartens with the lower wheezing syndrome prevalence (1-3^rd^ quartiles) (*N* = 1,319)	Kindergartens with the highest wheezing syndrome prevalence (4^th^ quartile) (*N* = 475)
% Males (*n*)		50.9 (890)	50.2 (645)	53.1 (245)
Mean age (± SD)		4.07 (± 1.44)	4.15 (± 1.4)	3.85 (± 1.49)^**^
Mean BMI (± SD)		15.52 (± 2.02)	15.50 (± 2.06)	15.57 (± 1.91)
Mean BMI–z score (± SD)		−0.0005 (± 1.00)	−0.0099 (± 1.02)	0.0255 (± 0.94)
% Diagnosed with asthma (*n*)		2.8 (47)	2.2 (27)	4.6 (20)^*^
% Have siblings (*n*)		61.5 (1,076)	59.6 (766)	66.8 (310)^*^
% Exposed to secondhand smoking (*n*)		33 (572)	33.9 (431)	30.5 (141)
% Diagnosed with eczema (*n*)		16.5 (272)	15.8 (193)	18.5 (79)
% Diagnosed with allergic rhinitis (*n*)		5.1 (87)	5.0 (62)	5.6 (25)
% Have a cat or a dog at home (*n*)		44.2 (766)	45.0 (572)	42.0 (194)
% Live next to the railway (*n*)		7.5 (130)	6.6 (84)	9.9 (46)^*^
% Have an air conditioner at home (*n*)		29.0 (506)	28.2 (360)	31.4 (146)
% Kindergarten next to railway (*n*)		33.7 (604)	22.1 (291)	65.9 (313)^**^
Housing type	% Private home (*n*)	31.8 (554)	29.3 (375)	38.5 (179)^**^
	% Apartment (*n*)	68.2 (1,188)	70.7 (903)	61.5 (286)
Type of stove	% Electric (*n*)	70.5 (1,226)	69.6 (888)	72.8 (338)
	% Gas (*n*)	29.5 (513)	30.4 (387)	27.2 (126)
Median PM_1_ concentration (Particles/cm^3^)		52 (29; 70)	46 (24; 64)	57 (34; 70)^**^
Median PM_2.5_ concentration (Particles/cm^3^)		53 (31; 71)	47 (25; 66)	58 (35; 72)^**^
Median PM_10_ concentration (Particles/cm^3^)		54 (31;71)	47 (26; 66)	59 (36; 72)^**^

^*^p < 0.05; ^**^p < 0.001.

Binary logistic regression models were constructed to identify the most important determinants independently associated with wheezing among preschool children (Table 4). At the individual level, male gender and diagnosis of allergic rhinitis and eczema significantly increased the risk of wheezing. A higher risk and greater prevalence of wheezing at the kindergarten level were associated with younger child age and the presence of siblings. Environmental factors played a significant role: the risk of wheezing was elevated in kindergartens located in closer proximity to a railway and in those with higher concentrations of PM_1_ and, in particular, PM_2.5_.

**Table 4:** Binary logistic regression models explaining possible determinants of wheezing syndrome prevalence.

	Experienced wheezing syndrome at least once during lifetime		Kindergartens with the highest wheezing syndrome prevalence (4^th^ quartile)	
Variables	OR_adj_ (95%CI)	*p*	OR_adj_ (95%CI)	*p*
Male gender (vs. females)	2.01 (1.27–3.17)	0.003		
Diagnosed with eczema (vs. no)	1.76 (1.07–2.89)	0.027		
Diagnosed with allergic rhinitis (vs. no)	4.48 (2.42–8.29)	< 0.001		
	*N* = 1,588; Hosmer-Lemeshow test chi^2^ = 0.139; *p* = 0.933; correctly classified 94.3%; Nagelkerke R^2^ = 0.063			
Kindergarten next to the railway (vs. no)			7.57 (5.69–10.07)	< 0.001
Age (increase per 1 year)			0.85 (0.78–0.92)	< 0.001
Have siblings (*vs*. no)			1.39 (1.07–1.79)	0.013
PM_1_ (per increase in particles/cm^3^)			1.79 (1.25–2.56)	0.001
PM_2.5_ (per increase in particles/cm^3^)			134.48 (52.75–342.89)	< 0.001
PM_10_ (per increase in particles/cm^3^)			0.004 (0.002–0.009)	< 0.001
			*N* = 1,736; Hosmer-Lemeshow test chi^2^ = 267.543; *p* < 0.001; correctly classified 73.4%; Nagelkerke R^2^ = 0.283	

## 4 Discussion

In this study, potential determinants of wheezing among preschool-aged children were investigated. Findings of current research indicate that wheezing is associated with the presence of other allergic diseases at the individual level. At the kindergarten level, a higher prevalence of wheezing was observed in facilities with elevated concentrations of particulate matter, as well as in those located in close proximity to railways.

The frequency of parent-reported respiratory symptoms among preschool children was assessed. Parent-reported wheezing is considered one of the most important criteria not only for identifying wheezing episodes but also for supporting the diagnosis of asthma in childhood (9, 19). Although the prevalence of physician-diagnosed asthma was relatively low (2.6%), the prevalence of at least one episode of wheezing during the lifetime (5.8%) and cough lasting two or more months (7.8%) was higher, indicating a greater burden of respiratory symptoms among preschool children. Parents reported that the prevalence of asthma and wheezing ever in children was lower than that previously observed among older Polish children, where it exceeded 8% and 20% of the study population, respectively (16). Other studies have also indicated that wheezing is one of the most common health disorders among preschool children (31, 32). The relatively low prevalence of wheezing observed may be attributable to the subjective and not always accurate parental perception of this symptom. Wheezing in preschool children is typically identified retrospectively based on parental reports; however, parents may not reliably differentiate wheezing from other respiratory sounds (19). In addition, the questionnaire included separate items on bronchiolitis, pneumonia, and other respiratory infections, which may have led parents to report these conditions instead of wheezing, thereby contributing to a lower reported prevalence of wheezing. Gender differences in the prevalence of respiratory symptoms were observed. Both asthma and wheezing were more common among boys than girls. However, only the difference in wheezing incidence was statistically significant. These findings are consistent with previous studies among school-aged children, which reported that both wheezing and asthma diagnoses were more prevalent among boys (16, 33). This study finding is consistent with previous research, indicating that children with siblings are at higher risk of wheezing due to viral infection transmission within the home environment (34).

Respiratory health in children is greatly influenced by the home environment and related exposures (5). Although direct measurements of particulate matter concentrations in the children's homes were not performed, the main home environmental exposures were assessed through the questionnaire and included in the subsequent analysis. Surprisingly, wheezing did not differ significantly between children exposed to common household risk factors, including second-hand smoke from living with smokers, third-hand smoke residues on surfaces (35), or having pets at home, and those without such exposures. Other studies have shown clear associations between these risk factors and recurrent or chronic wheeze, supporting the importance of smoking as a key factor for the development of a single symptom to chronic disease (36–39). In contrast, in this study preschool children preschool children were analyzed as a heterogeneous group with both recurrent/chronic and single episodes of wheeze, looking at the basic effects of aerosol pollution.

The results of this study were consistent with previous research, as the association between air pollution and respiratory disorders in children has been recognized and extensively investigated (5, 17, 24, 40). Both indoor and ambient air pollution are considered important contributors to adverse respiratory outcomes, with their effects largely mediated through inflammatory processes in the respiratory tract (20–22, 41). Nevertheless, the underlying biological mechanisms are not yet fully elucidated (42). However, evidence about the impact of air quality in kindergarten environments on younger children is still limited (3, 43), which highlights the importance of findings.

Multivariate analysis was performed to identify variables independently associated with wheezing syndrome in preschool children. Variables that were significantly associated with wheezing ever at the individual level and with wheezing prevalence at the kindergarten level were included in the multivariable analysis. Asthma diagnosis was excluded from the regression models due to multicollinearity with wheezing, as these variables were strongly correlated; notably, wheezing represents one of the principal clinical symptoms of asthma, further supporting their close interrelationship. In the overall study population, only male sex and a diagnosis of allergic diseases—such as asthma, eczema, or allergic rhinitis—were associated with a higher prevalence of wheezing and shortness of breath. However, when children were grouped according to kindergarten, environmental factors became more prominent, particularly PM_1_ and PM_2.5_ indoor concentrations and attendance at a kindergarten located near a railway. In contrast, an inverse association was observed with PM_10_ concentration. This finding may be explained by collinearity among particulate matter fractions, as PM_10_ encompasses both PM_1_ and PM_2.5_, potentially leading to overlapping effects within the multivariable model.

Findings of this study confirm that indoor air pollution is strongly influenced by outdoor air pollution, especially in urban areas where traffic and industrial pollution are among the main factors contributing to increased respiratory symptoms among children (26, 44, 45). It is also important to note that, kindergartens located near railways partly represent industrial areas, as railway infrastructure is often situated close to industrial zones.

Although both living near a railway and attending a kindergarten located near a railway were significantly associated with a higher risk of wheezing in the univariate analysis, only the kindergarten's location remained a significant predictor in the multivariate analysis. It indicates that air quality in the kindergarten environment is at least as important as air quality at home and may even have a stronger influence on children's respiratory health.

The study demonstrates that not only PM_2.5_, but also finer particulate matter in the PM_1_ fraction (0.3–1.0 μm), is significantly associated with wheezing syndrome in preschool-aged children. From previous research it is already known the association between PM_1_ exposure and physician-diagnosed asthma in younger school-aged children (7). Taken together, these findings provide indirect evidence that wheezing syndrome in early childhood and asthma share more common than distinct etiopathogenetic mechanisms. Moreover, in certain cases, the first episode of wheezing may represent the earliest clinical manifestation of asthma.

The study's results suggest that additional attention should be given to air quality in kindergartens. This is supported by previous studies showing higher rates of respiratory symptoms among children exposed to traffic- and industry-related pollution, construction and renovation works, coal burning, and household smoking (26, 46). Indoor air quality in kindergartens is also influenced by factors such as occupancy density, occupant behavior, ventilation practices, and routine activities including cooking and cleaning (47).

Accordingly, preventive actions should be implemented at the individual, local, and national levels to better control environmental factors and protect children's health, including better planning for building location and building design, ensuring proper ventilation in kindergartens‘ canteens (43), designed barriers of plants near the kindergartens (i.e., in terms of species, leaf density, permeability, height) installed to reduce particulate matter concentrations outdoor (30, 48), using mechanical ventilation in separate, in old buildings poorly designed spaces, such as those where parents' meetings are held or in a poorly designed gym, and developing and implementing indoor air quality guidelines (28).

This study has both strengths and limitations. The strength of the presented study is the large study population, which increases statistical power and enhances the generalizability of the findings. Second, this study integrates direct measurements of air pollution in kindergarten environments with the assessment of respiratory symptom prevalence among preschool children, thereby addressing an important gap in the existing literature (49). In addition, previously conducted several studies in school settings (10, 24, 25), provides a broader scientific background in this field. This continuity offers important opportunities for future research to compare data from schools and kindergartens, enabling a more comprehensive understanding of the origins and development, as well as the prevention, of chronic non-communicable diseases in childhood.

The limitation of this study is its cross-sectional design. Although such a design enables the assessment of relationships between air pollution and wheezing, it does not allow for the establishment of causality. Another limitation of the study is that the questionnaires were self-administered by parents, which may have introduced response bias, and a proportion of questionnaires were not returned. Due to a lack of video demonstrations of different wheeze patterns, parents may misinterpret questions about wheezing, thereby underestimating the rate of children affected. Future studies could improve data completeness and accuracy by employing interviewer-administered surveys. Finally, the home environment was assessed solely through a parent-completed questionnaire, while no objective indoor air pollution measurements were conducted. The incorporation of clinical assessments and biological markers reflecting cumulative environmental exposure could strengthen future research in this area.

## 5 Conclusions

Indoor exposure to particulate matter in kindergartens was more strongly associated with wheezing in preschoolers than common household risk factors, such as pets or passive tobacco smoke. A higher prevalence of wheezing was observed in kindergartens with elevated PM_1_ and PM_2.5_ concentrations and in those located close to railways. These findings highlight that the kindergarten environment where children spend a large part of their day, may substantially affect respiratory morbidity. Therefore, improving indoor air quality in kindergartens through effective control of air pollutants, installation of vegetative barriers near kindergartens that promote particulate matter dry deposition from outdoor air pollution sources, and adherence to indoor air quality guidelines are essential for preventing recurrent wheeze in early childhood.

## Data Availability

The raw data supporting the conclusions of this article will be made available by the authors, without undue reservation.

## References

[1] Azam NSA Jalaludin J Suhaimi NF. The association between indoor air quality and respiratory health symptoms among preschool children in Penang, Malaysia. Int J Environ Health Res. (2025) 35:606–19. doi: 10.1080/09603123.2024.2365308 38860645

[2] Valiulis A Bousquet J Veryga A Suprun U Sergeenko D Cebotari S . Vilnius declaration on chronic respiratory diseases: multisectoral care pathways embedding guided self-management, mHealth and air pollution in chronic respiratory diseases. Clin Transl Allergy. (2019) 9:1–10. doi: 10.1186/s13601-019-0242-2 30705747 PMC6348633

[3] Zakaria IB Mahyuddin N. An overview of indoor air pollution in the Malaysian kindergarten environment. In: IOP Conference Series: Earth and Environmental Science 2022. IOP Publishing. (2022) 1013:012005. doi: 10.1088/1755-1315/1013/1/012005

[4] Aithal SS Sachdeva I Kurmi OP. Air quality and respiratory health in children. Breathe. (2023) 19:230040. doi: 10.1183/20734735.0040-2023 37377853 PMC10292770

[5] Lee JX. Gaffin, JM. Recent evidence on indoor air pollutants and pediatric asthma morbidity. Pediatr Pulm. (2026) 61:e71483. doi: 10.1002/ppul.71483 41608962

[6] Sauliene I Valiulis A Keriene I Sukiene L Dovydaityte D Prokopciuk N . Airborne pollen and fungi indoors: evidence from primary schools in Lithuania. Heliyon. (2023) e9:e12668. doi: 10.1016/j.heliyon.2022.e12668 PMC9850001 36685406

[7] Juskiene I Prokopciuk N Franck U Valiulis A Valskys V Mesceriakova V . Indoor air pollution effects on pediatric asthma are submicron aerosol particle-dependent. Eur J Pediatr. (2022) 181:2469–80. doi: 10.1007/s00431-022-04443-6 35312840

[8] Su JG Aslebagh S Shahriary E Barrett M Balmes JR. Impacts from air pollution on respiratory disease outcomes: a meta-analysis. Front Public Health. (2024) 12:1417450. doi: 10.3389/fpubh.2024.1417450 39444957 PMC11497638

[9] Bush A. Basic clinical management of preschool wheeze. Pediatr Allergy Immunol. (2023) 34:e13988. doi: 10.1111/pai.13988 37492909

[10] Prokopciuk N Franck U Dudoitis V Tarasiuk N Juskiene I. Cepuraite, et al. Global alliance against chronic respiratory diseases demonstration project: aerosol pollution and its seasonal peculiarities in primary schools of Vilnius. Chin Med J. (2020) 133:1516–25. doi: 10.1097/CM9.0000000000000913 32568873 PMC7386335

[11] USEPA. Reference Guide for Indoor Air Quality in Schools. (2025). https://www.epa.gov/iaq-schools/reference-guide-indoor-air-quality-schools#Introduction [Accessed March 18, 2026].

[12] Maung TZ Bishop JE Holt E Turner AM Pfrang C. Indoor air pollution and the health of vulnerable groups: a systematic review focused on particulate matter (PM), volatile organic compounds (VOCs) and their effects on children and people with pre-existing lung disease. Int J Environ Res Public Health. (2022) 19:8752. doi: 10.3390/ijerph19148752 35886604 PMC9316830

[13] Morawska L Ayoko GA Bae GN Buonanno G Chao CYH Clifford S . Airborne particles in indoor environment of homes, schools, offices and aged care facilities: the main routes of exposure. Environ Int. (2017) 108:75–83. doi: 10.1016/j.envint.2017.07.025 28802170

[14] Mei SD Luen LC. Effect of kindergarten environment creation quality on children health, language, social, science and art. Int J Acad Res Prog Educ Dev. (2023) 12:187. doi: 10.6007/IJARPED/v12-i2/16714

[15] Midouhas E Kokosi T Flouri E. Outdoor and indoor air quality and cognitive ability in young children. Environ Res. (2018) 161:321–8. doi: 10.1016/j.envres.2017.11.026 29182908

[16] Wypych-Slusarska A Grot M Kujawińska M Nigowski M Krupa-Kotara K. Oleksiuk et al. Respiratory symptoms, allergies, and environmental exposures in children with and without asthma. Int J Environ Res Public Health. (2022) 19:11180. doi: 10.3390/ijerph191811180 36141448 PMC9517590

[17] Holst GJ Pedersen CB Thygesen M Brandt J Geels C . Air pollution and family related determinants of asthma onset and persistent wheezing in children: nationwide case-control study. BMJ. (2020) 370:m2791. doi: 10.1136/bmj.m2791 32816747 PMC7437497

[18] Brand PLP Baraldi E Bisgaard H Boner AL Castro-Rodriguez JA Custovic A . Definition, assessment and treatment of wheezing disorders in preschool children: an evidence-based approach. Eur Respir J. (2008) 32:1096–110. doi: 10.1183/09031936.00002108 18827155

[19] Makrinioti H Fainardi V Bonnelykke K Custovic A Cicutto L Coleman C . European respiratory society statement on preschool wheezing disorders: updated definitions, knowledge gaps and proposed future research directions. Eur Respir J. (2024) 64:2400624. doi: 10.1183/13993003.00624-2024 38843917

[20] Sly PD. Adverse environmental exposure and respiratory health in children. Pediatr Clinic. (2021) 68:277–91. doi: 10.1016/j.pcl.2020.09.018 33228938

[21] Keleb A Abeje ET Daba C Endawkie A Tsega Y Abere G . The odds of developing asthma and wheeze among children and adolescents exposed to particulate matter: a systematic review and meta-analysis. BMC Public Health. (2025) 25:1225. doi: 10.1186/s12889-025-22382-3 40165124 PMC11959839

[22] Tsai YG Liu CS Hung CH Yang HY Yeh YP Chang YJ . Asthmatic symptoms in schoolchildren: effect of PM2.5 exposure, oxidative stress, and lung function growth. Pediatr Res. (2025) 1–9. doi: 10.1038/s41390-025-04454-7 41345332

[23] Eurydice Network. Key data on early childhood education and care in Europe 2025. Luxembourg: Publications Office of the European Union (2025). 236 p.

[24] Prokopciuk N Taminskiene V Vaideliene L Juskiene I Svist V Valiulyte I . The incidence of upper respiratory infections in children is related to the concentration of vanadium in indoor dust aggregates. Front Public Health. (2024) 12:1339755. doi: 10.3389/fpubh.2024.1339755 38577275 PMC10993999

[25] Taminskiene V Prokopciuk N Karvelyte V Vaitkaitiene E Butikis M Valiulis A . A cross-sectional analysis of air pollution in primary schools and children's fatigue. Front Public Health. (2025) 13:1595089. doi: 10.3389/fpubh.2025.1595089 40959634 PMC12434117

[26] Liu MM Wang D Zhao Y Liu YQ Huang MM Liu Y . Effects of outdoor and indoor air pollution on respiratory health of Chinese children from 50 kindergartens. J Epidemiol. (2013) 23:280–87. doi: 10.2188/jea.JE20120175 23728483 PMC3709542

[27] Biswas S Elma SI Kabir A. Indoor air quality and possible health risks at kindergarten schools in megacities: evidence from Bangladesh. Water Air Soil Pollut. (2025) 236:876. doi: 10.1007/s11270-025-08511-z

[28] Błaszczyk E Rogula-Kozłowska W Klejnowski K Kubiesa P Fulara I. Mielżyńska-Švach, D. Indoor air quality in urban and rural kindergartens: short-term studies in Silesia, Poland. Air Qual Atmos Health. (2017) 10:1207–20. doi: 10.1007/s11869-017-0505-9 29308098 PMC5741794

[29] Abdel-Salam MM. Seasonal variation in indoor concentrations of air pollutants in residential buildings. J Air Waste Manag Assoc. (2021) 71:761–77. doi: 10.1080/10962247.2021.1895367 33625321

[30] Sheikh HA Maher BA Woods AW Tung PY. Harrison RJ. Efficacy of green infrastructure in reducing exposure to local, traffic-related sources of airborne particulate matter (PM). Sci Total Environ. (2023) 903:166598. doi: 10.1016/j.scitotenv.2023.166598 37634712

[31] Alqwaiee M Al-Harbi AS. Wheezing in children: approaches to diagnosis and management. Int J Pediatr Adolesc Med. (2019) 6:68–73. doi: 10.1016/j.ijpam.2019.02.003 31388550 PMC6676316

[32] Song P Adeloye D Salim H Dos Santos JP Campbell H Sheikh A . Global, regional, and national prevalence of asthma in 2019: a systematic analysis and modelling study. J Glob Health. (2022) 12:04052. doi: 10.7189/jogh.12.04052 35765786 PMC9239324

[33] Trivedi M Denton E. Asthma in children and adults—what are the differences and what can they tell us about asthma? Front Pediatr. (2019) 7:256. doi: 10.3389/fped.2019.00256 31294006 PMC6603154

[34] Lisik D Ioannidou A Milani GP Nyassi S Ermis SÖ Spolidoro GCI . Birth order, sibship size and wheezing phenotypes: a systematic review and meta-analysis. Eur Respir J. (2022) 60:1530. doi: 10.1183/13993003.congress-2022.1530

[35] Acuff L Fristoe K Hamblen J Smith M Chen J. Third-hand smoke: old smoke, new concerns. J Community Health. (2016) 41:680–87. doi: 10.1007/s10900-015-0114-1 26512014

[36] Taminskiene V Mukhopadhyay S Palmer C Mehta A Ayres J Valiulis A . Factors associated with quality of life in children with asthma living in Scotland. Pediatr Pulmonol. (2016) 51:484–90. doi: 10.1002/ppul.23359 26669689

[37] Ji X Yao Y Zheng P Hao C. The relationship of domestic pet ownership with the risk of childhood asthma: a systematic review and meta-analysis. Front Public Health. (2022) 10:953330. doi: 10.3389/fped.2022.953330 PMC9352935 35935350

[38] Ali S Raza MA Fazal S Ahmad RT Fatima M . Exploring the association between passive exposure to household smoking and frequency of recurrent wheezing in children. JHWCR. (2025) e308–e308. doi: 10.61919/wj7bc393

[39] Bush A Ferkol T Valiulis A Mazur A Chkhaidze I Maglakelidze T . Unfriendly fire: how the tobacco industry is destroying the future of our children. Acta med Litu. (2021) 28:6–18. doi: 10.15388/Amed.2020.28.1.6 34393624 PMC8311841

[40] Bonato M Gallo E Bazzan E Marson G Zagolin L Cosio MG . Air pollution relates to airway pathology in children with wheezing. Ann Am Thorac Soc. (2021) 18:2033–40. doi: 10.1513/AnnalsATS.202010-1321OC 34004126 PMC8641808

[41] Hua L Ju L Xu H Li C Sun S Zhang Q . Outdoor air pollution exposure and the risk of asthma and wheezing in the offspring. Environ Sci Pollut Res. (2023) 30:14165–89. doi: 10.1007/s11356-022-23094-6 36149565

[42] Altman MC Kattan M. T O'Connor G, Murphy RC, Whalen E, LeBeau P, et al. Associations between outdoor air pollutants and non-viral asthma exacerbations and airway inflammatory responses in children and adolescents living in urban areas in the USA: a retrospective secondary analysis. Lancet Planet Health. (2023) 7:e33–44. doi: 10.1016/S2542-5196(22)00302-3 36608946 PMC9984226

[43] Zakaria IB Mahyuddin N Mohd-Sahabuddin MF. Change of use challenges: unveiling indoor air quality in converted kindergarten buildings. Int J Environ Res. (2025) 19:89. doi: 10.1007/s41742-025-00756-0

[44] Ródenas García M Spinazzé A Branco PT Borghi F Villena G Cattaneo A . Review of low-cost sensors for indoor air quality: features and applications. Appl Spectrosc Rev. (2022) 57:747–79. doi: 10.1080/05704928.2022.2085734

[45] Paciência I Cavaleiro Rufo J Moreira A. Environmental inequality: air pollution and asthma in children. Pediatr Allergy Immunol. (2022) 33:e13818. doi: 10.1111/pai.13818 35754123

[46] Prokopciuk N Juskiene I Tarasiuk N Franck U Kostiuk O Valiulis A . On the additional risk for human health in the use of sandblasting of building walls. Environ Sci Pollut Res. (2023) 30:56558–68. doi: 10.1007/s11356-023-26382-x 36920615

[47] Zakaria IB Mahyuddin N Mohd-Sahabuddin MF. Factors influencing indoor air pollution in kindergarten: a systematic literature review. ARASET. (2026) 55:154–74. doi: 10.37934/araset.55.2.154174

[48] Greenwald R Sarnat JA Fuller CH. The impact of vegetative and solid roadway barriers on particulate matter concentration in urban settings. PLoS ONE. (2024) 19:e0296885. doi: 10.1371/journal.pone.0296885 38295020 PMC10830032

[49] Anake WU Nnamani EA. Indoor air quality in day-care centres: a global review. Air Qual Atmos Health. (2023) 16:997–1022. doi: 10.1007/s11869-023-01320-5 36819788 PMC9930043

