# Supplementary material for: Indoor air pollution in kindergartens is a stronger predictor of preschool wheezing than household pets and passive smoking
Source: Front Public Health. 2026 May 29;14:1835292. doi: 10.3389/fpubh.2026.1835292 (PMC13260636; doi:10.3389/fpubh.2026.1835292)
Supplement: Supplementary file 1 [file Table_1.docx]

**This questionnaire is filled out by the parents.** Please answer the questions as shown in the example.

6

**Example:** If required, write your answer in the box provided: Age

By answering yes/no, please tick the appropriate answer box.

No

Yes

√

____________________________________________________________________________________________

These questions are about your child’s health and their environment. Please write your answers in the provided fields.

Day

Month

Years

Date of completion:

Years

Month

Day

Child‘s birth date:

How many months have you been attending this kindergarten?

Girl

Boy

Child‘s gender: Child‘s height cm Child‘s weight kg

Name of kindergarten:

Does the child have siblings growing up together? If yes, please indicate their gender and age in the field below:

NO

YES

**Questions about eczema**

1. Has your child ever had an itchy rash that lasted for at least 6 months? IF YOU ANSWERED “NO”, CONTINUE FROM QUESTION 7.

NO

YES

2. Has your child ever had an itchy rash in the past 12 months? IF YOU ANSWERED “NO”, CONTINUE FROM QUESTION 7.

NO

YES

3. Has this rash ever appeared in any of the following areas: the folds of the elbows, behind the knees (popliteal areas), the front of the ankles, under the buttocks, on the face, cheeks, or around the neck, ears, or eyes?

YES

NO

4. How old was the child when this rash first appeared? Write down the year and month if you remember.

Years

Months

5. Has this rash completely disappeared for any period of time in the past 12 months?

YES

NO

6. In the past 12 months, how often did your child not sleep at night (wake up, slept poorly) because of itching?

Never during the last 12 months

1 or more nights per week

Less than 1 night per week

1. Has your child ever been diagnosed with atopic dermatitis (eczema) by a physician?

NO

YES

8. If your child has been diagnosed with atopic dermatitis and you know its severity score according to SCORAD, enter it.

SCORAD now

SCORAD at diagnosis

Unknown

**Questions about other allergies**

**Asthma**

1. Has your child ever experienced shortness of breath or wheezing in the chest? IF YOU ANSWERED ‘NO’, CONTINUE ANSWERING FROM QUESTION 6

YES

NO

1. During the past 12 months, has your child experienced shortness of breath or wheezing in the chest, or had episodes of breathlessness? IF YOU ANSWERED ‘NO’, CONTINUE ANSWERING FROM QUESTION 6.

YES

NO

1. How many times in the past 12 months has the child experienced shortness of breath or sudden coughing fits?

More than 12

4-12

1-3

None

1. How many times on average in the past 12 months did the child wake up due to shortness of breath or paroxysmal coughing??

Less than 1 night per week

One or more nights per week

Never

1. During the past 12 months, was the shortness of breath so severe that the child could only say one or two words between breaths?

YES

NO

1. Has your child ever been diagnosed with asthma by a physician?

NO

YES

1. If yes, what type?

Non- allergic

Allergic

Non-specified

1. If your child has been diagnosed with asthma, please indicate its severity level, if known.

Moderate

Severe

Mild

Not known

1. During the past 12 months, has the child had a dry cough at night without having a cold, flu, or respiratory infection?

YES

NO

1. Has your child had a cough lasting longer than two months?

NO

YES

**Allergic rhinitis**

1. Has your child ever sneezed, had a runny nose, or had a stuffy nose even when they didn't have a cold or the flu? IF YOU ANSWERED ‘NO’, CONTINUE ANSWERING FROM QUESTION 6.

YES

NO

1. In the past 12 months, has your child had sneezing, runny nose, or a stuffy nose even when they didn't have a cold or flu? IF YOU ANSWERED ‘NO’, CONTINUE ANSWERING FROM QUESTION 6.

NO

YES

1. Have the symptoms listed in the first question been accompanied by itchy-watery eyes in the past 12 months?

NO

YES

1. In which months did your child experience these symptoms? Please check all months that apply.

February

January

March

June

April

May

December

November

October

September

August

July

5. To what extent have the symptoms listed in the first question of this section been related to your child's daily activities over the past 12 months?

Somewhat

Not related

Moderate

Slightly

1. Has your child ever been diagnosed with allergic rhinitis by a physician?

NO

YES

1. If allergic rhinitis was diagnosed, what was the severity level determined by the doctor?

Severe

Moderate

Mild

Unknown

**Questions about respiratory infection**

1. How many times on average per year (estimated over the past 2 years) has your child had acute upper respiratory tract infections?

>1 time per month

None

1 time per month

1 time per 3 months

1 time per year

2. Has your child had pneumonia in the last 12 months?

YES

NO

1. Can you tell that every cold your child has ends in a lower respiratory tract infection?

YES

NO

**Has your child ever been diagnosed with one of the following conditions by a doctor? If so, please indicate how many times**

1. Cold (acute runny nose, acute sore throat)

Times

YES

NO

1. Bronchitis

Times

YES

NO

1. Obstructive bronchitis

Times

YES

NO

1. Bronchiolitis (shortness of breath or noisy breathing in a child under 2 years old with a fever)

Times

YES

NO

1. Stenosing laryngitis (croup)

Times

YES

NO

1. Otitis media

Times

YES

NO

1. Sinusitis

Times

YES

NO

1. Adenoiditis (enlargement of adenoids, polyps, requiring treatment)

Times

YES

NO

**Questions about allergy tests performed**

1. Have allergy tests been performed?

NO

YES

1. If yes, what tests have been performed? Check all tests performed. If no tests have been performed, check the appropriate box.

Skin prick samples

Specific IgE

Total IgE

1. Positive results for food allergens. Check all that apply. If there is no suitable option, enter your own.

Other (please specify)

Egg white

Not detected

Wheat

Milk

1. Positive results for inhaled allergens. Check all that apply. If no suitable option is available, enter your own.

Grass, weed pollen

House dust mites

Tree pollen

Other (please specify)

Not detected

Animals

**Questions about environment**

1. What is the distance from your place of residence to the nearest highway?

≈50 m or less

≈ 50 –200 m

≈ 200 m or more

1. How often per day do trucks/lorry vehicles pass near your home?

All the time

Several times a week

Every day

Several times a day

Once a week per savaitę

Never

1. Do you live near a railway (or is it visible from your home windows)?

YES

NO

1. During the child’s first year of life, did you keep a cat and/or a dog in the home?

NO

YES

1. During the past 12 months, have you kept a cat and/or a dog in your home?

NO

YES

1. Are there people who smoke in the child’s environment (including electronic cigarettes)?

NO

YES

1. If yes, what type of cigarettes?

There are no smokers

Electronic

Tobacco al

1. Do grandparents, cousins, uncles, or aunts of the child live together with you?

NO

YES

1. Is there an air conditioner in your home?

YES

NO

1. Do you use it only in summer (during warm period)?

There is no air conditioner

NO

YES

1. What is the type of residential building?

Private house

Appartment

1. If you live in an apartment, which floor? Write the floor number in the box.

1. If you live in a private house, what is the type of heating?

Gass

Other (fill-in)

Geothermal

Central

Electricity

Solid fuel

1. What type of stove do you use for cooking?

Solid fuel

Gass

Electric

1. Please enter the postal code of your place of residence for an assessment of environmental pollution based on the data of the nearest pollution monitoring station.

**Thank you for participating in the survey. If you agree to be contacted by a researcher for clarification of your answers, please provide your email address or phone number.**
